# From licensure to residency: structural barriers and policy options in Israel’s residency admission process

**DOI:** 10.1186/s13584-026-00774-z

**Published:** 2026-09-02

**Authors:** Ayala Burger, Alexey Belinsky, Joseph Mendlovic, Daniel Minerbi Padon, Michal Laron

**Affiliations:** 1 Myers-JDC-Brookdale Institute, JDC Hill, Jerusalem, Israel; 2 Ministry of Health, Jerusalem, Israel; 3 Shaare Zedek Medical Center, Affiliated with the Hadassah-Hebrew University School of Medicine, Jerusalem, Israel; 4 Ministry of Finance, Jerusalem, Israel

**Keywords:** Residency, Medical workforce planning, Matching, Israel, Health policy, Medical education

## Abstract

**Background:**

Israel faces a growing need for medical professionals due to population growth and aging, yet the number of licensed physicians per capita remains below the OECD average. The gap is particularly evident in the number of medical specialists. One factor contributing to this shortage may be the delay between medical licensure and the commencement of residency training.

**Objective:**

The objective of this study was to assess the extent to which delays in the commencement of residency programs occur, to identify and analyze barriers that delay the start of residency training among newly licensed physicians in Israel, and to explore possible solutions to optimize this process.

**Methods:**

This mixed-methods study included a quantitative analysis of administrative datasets from the Israeli Ministry of Health (*n* = 18,480), and qualitative analysis based on 31 semi-structured interviews conducted with department heads, residents, and residency candidates between January and August 2023.

**Results:**

Despite a steady increase in the number of licensed physicians, the median time between licensure and the start of residency has remained constant at approximately four months. According to interviewees’ accounts in the study, delays were often associated with inconsistent and opaque admission processes, regional disparities in hospital demand, and mismatched expectations between candidates and departments, and could in part be explained by the absence of a centralized residency allocation system. Factors such as elective choices during internship and geographic location further influenced the timeline. Interviewees did not commonly report postponing residency for personal reasons.

**Conclusions:**

Delays in residency placement, compounded by inefficient workforce allocation, may reduce the effective capacity of the healthcare system. A more transparent and structured national framework - similar to those in the US, Canada, and Spain - could help streamline the process and reduce inefficiencies. Policy recommendations include improved data transparency, standardization of admission criteria, and better support for hospitals in peripheral regions. Establishing a unified, annual data reporting system on the physician workforce is a necessary prerequisite for any effective reform or national workforce planning.

## Background

Israel’s population is growing steadily, with the highest fertility rate among OECD countries (3.1 compared to an OECD average of 1.58) and a rising proportion of older adults (OECD, [13, 18]). These demographic trends are expected to increase the demand for medical services. At the same time, Israel’s physician-to-population ratio (3.3 per 1,000 people) remains below the OECD average of 3.6 (OECD, [14]), and a significant proportion of physicians are nearing retirement - by 2021, a quarter of licensed physicians (*n* = 43,262) were aged 67 or older [5].

The physician training pathway is long and complex. It consists of multiple stages leading up to final certification as a specialist physician. The beginning of the physician training pathway in Israel is medical school, in a six-year or four-year track (for first-degree graduates). This is followed by the internship period (staž). The placement of the internship period is conducted through a lottery organized by the Ministry of Health. In this period, there are mandatory rotations of several months and elective rotations of one month in departments chosen by the students. After completing these stages and successfully passing the final exams for each rotation, physicians trained in Israel receive their Israeli medical license. Graduates of foreign medical studies must take a government licensing exam. Upon passing this exam, and successfully completing the internship year, they are authorized to practice medicine in Israel.

Following licensure, graduates may pursue a residency of their choice in a hospital department or clinic that accepts them. Unlike the internship placement, the search for residency is entirely the candidate’s responsibility. This process may take months or even years. During this time, the physician may remain outside of the clinical system. Some of the graduates awaiting acceptance into residency positions are employed as general practitioners in community urgent care centers or in other medical roles. However, no comprehensive data are currently available regarding their numbers or the trends in this employment pattern. This gap may represent a loss for both the system and the individual, as those months or years are rarely “made up” later in the career span. Delays over a year were identified by healthcare system officials as especially problematic and were used as a threshold in this study.

Alongside the factors described above, the shortage of medical personnel could be mitigated by shortening the duration between receiving an Israeli medical license and the actual commencement of residency training. Reducing this wait time would inherently decrease the medical workforce shortage by extending the overall period during which physicians are active within the clinical system. When a residency candidate spends an extended period searching for a position, the system loses a trainee who could have begun their service at an earlier date. In effect, a delay of several months or more translates into an equivalent loss of specialist labor later in the physician’s career. These lost months or years are unlikely to be recovered in the future, as there is no evidence to suggest a correlation between the residency start date and the age of retirement from the medical profession. Consequently, any systemic ambiguity or delay, as detailed below, adversely affects both the individual physician and the healthcare system as a whole.

Furthermore, a delay of several months is not the most detrimental scenario for the system; the frustration resulting from the inability to secure a residency placement may lead some physicians to seek training opportunities abroad or to pursue alternative career paths where they can utilize their medical knowledge outside of clinical practice. While the current study’s data do not allow for a precise estimation of the number of physicians who abandon the profession after licensure, it is reasonable to hypothesize that delays between licensure and residency entry increase the risk of the system permanently ‘losing’ these physicians as they progress in their careers.

Countries have adopted national systems to manage and match candidates to residency positions. Notable examples include the National Resident Matching Program (NRMP) in the United States [9], the Canadian Resident Matching Service (CaRMS) in Canada [17], and the Sistema Nacional de Residencias Médicas (SNRM) in Spain [3], which uses a nationwide exam (MIR) and centralized allocation based on exam score and preference ranking. Israel currently lacks such a national mechanism, leaving individual departments and candidates to navigate the process independently.

In Who Gets What - and Why, Alvin E. Roth develops a framework for diagnosing failures in matching markets by focusing on several structural dimensions. Market thickness refers to the extent to which participants interact in a single, coordinated marketplace. When thickness is low, positions go unfilled, anxiety rises, and lower-prestige institutions are systematically disadvantaged. Congestion arises when too many decentralized interactions occur without effective processing mechanisms, overwhelming participants and generating mismatches despite potentially good pairings. Roth also highlights the lack of safety in preference expression, which leads participants to behave strategically and rely on informal networks rather than reveal true preferences. These dynamics contribute to unraveling, as offers and commitments move to earlier, less informative stages, increasing instability and undermining trust. Finally, failures of transparency and entrenched norms further entrench inequities and constrain the design of effective centralized matching mechanisms, thereby delimiting feasible reform options alongside technical market design considerations [15]. Others highlight several structural disadvantages of centralized residency matching systems. They have been criticized for generating applicant anxiety and strategic behavior, as candidates feel limited control over outcomes and engage in signaling practices that undermine transparency [2] and violating the match rules [16]. Research also highlights systemic inefficiencies, including widespread over‑application and interview cancellations that burden both programs and applicants [12]. Additionally, structural critiques argue that the match functions as a monopsonistic labor market, restricting residents’ bargaining power and limiting their ability to negotiate or change positions [4].

As part of the efforts to address the anticipated shortage of physicians and to enable more effective workforce planning, the Ministry of Health established, for the first time, a national registry for medical residents. The registry, which became operational in 2023, made it possible for the first time to conduct the present study. This newly available dataset enables a uniquely broad empirical contribution: the first nationwide, twenty‑year analysis of the transition from licensure to residency. This study focused primarily on the residency search stages—the residency candidate’s considerations in deciding where and in which field to specialize and characterize the barriers and factors that delay the start of residency.

## Methods

This study employed a mixed-methods design, combining quantitative analysis of national administrative data with qualitative insights from semi-structured interviews. Together, these approaches enabled a comprehensive understanding of the barriers affecting the transition from licensure to residency training in Israel.

### Quantitative component

We analyzed data from the Israeli Ministry of Health, covering 18,480 physicians who were granted a medical license between 2000 and 2022. The dataset included demographic details (gender, age, place of residence, population group), country and institution of medical education, and date of licensure.

This dataset was merged with a second administrative file covering 15,116 physicians who began a residency in Israel (“residency registry”), which included dates of residency start and end, specialty, and number of residencies per physician. The merged dataset enabled calculation of time intervals between licensure and the beginning of residency. Physicians who commenced a residency and later discontinued their training or switched to a different specialty were included in the study population as having entered residency, as the primary focus was the initial transition from licensure.

#### Exclusions

Immigrant physicians were excluded due to differences in training pathways. Graduates of the military academic track (“Atuda”) were also excluded. Only first entry into residency was analyzed. Subsequent specialty changes and second residencies were not examined as separate outcomes.

#### Statistical analysis

Cross-tabulations and stratified analyses were used to examine relationships between variables. Proportion differences were tested using the Z-test. Differences in means of continuous variables were tested using t-tests. All analyses were conducted using IBM SPSS Statistics, Version 28.

### Qualitative component

A total of 31 semi-structured interviews were conducted between January and August 2023, mostly via Zoom, with two interviews held in person.

#### Interviewees included

8 department heads or deputy hospital directors involved in resident recruitment, 1 senior HR officer involved in residency staffing, 10 current residents in early years of training, and 12 physicians waiting to start residency.

#### Sampling strategy

Snowball sampling was used to identify interviewees. Participants were selected to reflect diversity in gender, ethnicity (Jewish, Arab, Druze), medical education location (Israel, approved foreign institutions, and unapproved foreign institutions^1^), and residency wait time (up to one year vs. one year or more). Interviewees came from eight hospitals and three of the four Israeli health maintenance organizations (HMOs).

#### Interview process

Senior physician interviews lasted approximately 1 h; resident/applicant interviews lasted approximately 30 min. Tailored interview protocols were used for each group. Interviews were recorded, transcribed, and coded using Atlas.ti 8. Thematic analysis was conducted by two independent researchers using thematic coding, followed by consensus discussions to ensure inter-rater reliability.

### Ethics

The study was approved by the Myers-JDC-Brookdale Institute Ethics Committee. Participation was voluntary. Interviewees were informed of their rights and gave oral consent. Only the research team had access to the secured data files.

## Results

### Characteristics of licensed physicians (2000–2022)

The dataset included 18,480 physicians who received their Israeli medical license between 2000 and 2022. Their key demographic and educational characteristics are presented in Table 1. The study population comprised 39.2% female physicians. The average age at license receipt was 30.6 years, with the vast majority of license recipients (88.5%) aged 26.0-34.9. Regarding population groups, 58.9% were Jewish, 38.1% Arab, and 1.6% Druze. Geographically, 24.8% of physicians lived in the Northern District, 22.3% in Tel Aviv District, and 19.0% in Central District. Physicians residing in the Jerusalem and Southern districts accounted for 10.9% and 8.9% of the study population, respectively. In terms of medical education, 51.1% of physicians studied at universities in Israel.

**Table 1:** Socio-demographic characteristics of recipients of Israeli medical license, 2000–2022 (percentages unless otherwise noted, *N* = 18,480)

Background Characteristic		%
Gender	Female	39.2
	Male	60.8
Population group	Jewish	58.9
	Arab	38.1
	Druze	1.6
	Unknown/Other	1.4
Age at license receipt	Mean (SD)	30.6 (3.7)
Age group at license receipt	< 26	1.1
	26–29	40.3
	30–34	48.2
	35–39	8.3
	≥ 40	2.2
District of residence	Jerusalem	10.9
	North	24.8
	Haifa	12.5
	Center	19.0
	Tel Aviv	22.3
	South	8.9
	The West Bank	1.6
Place of study	Universities in Israel	51.1
	Foreign study institutions	48.9

### Trends in medical licensure and residency entry

**Fig. 1:**
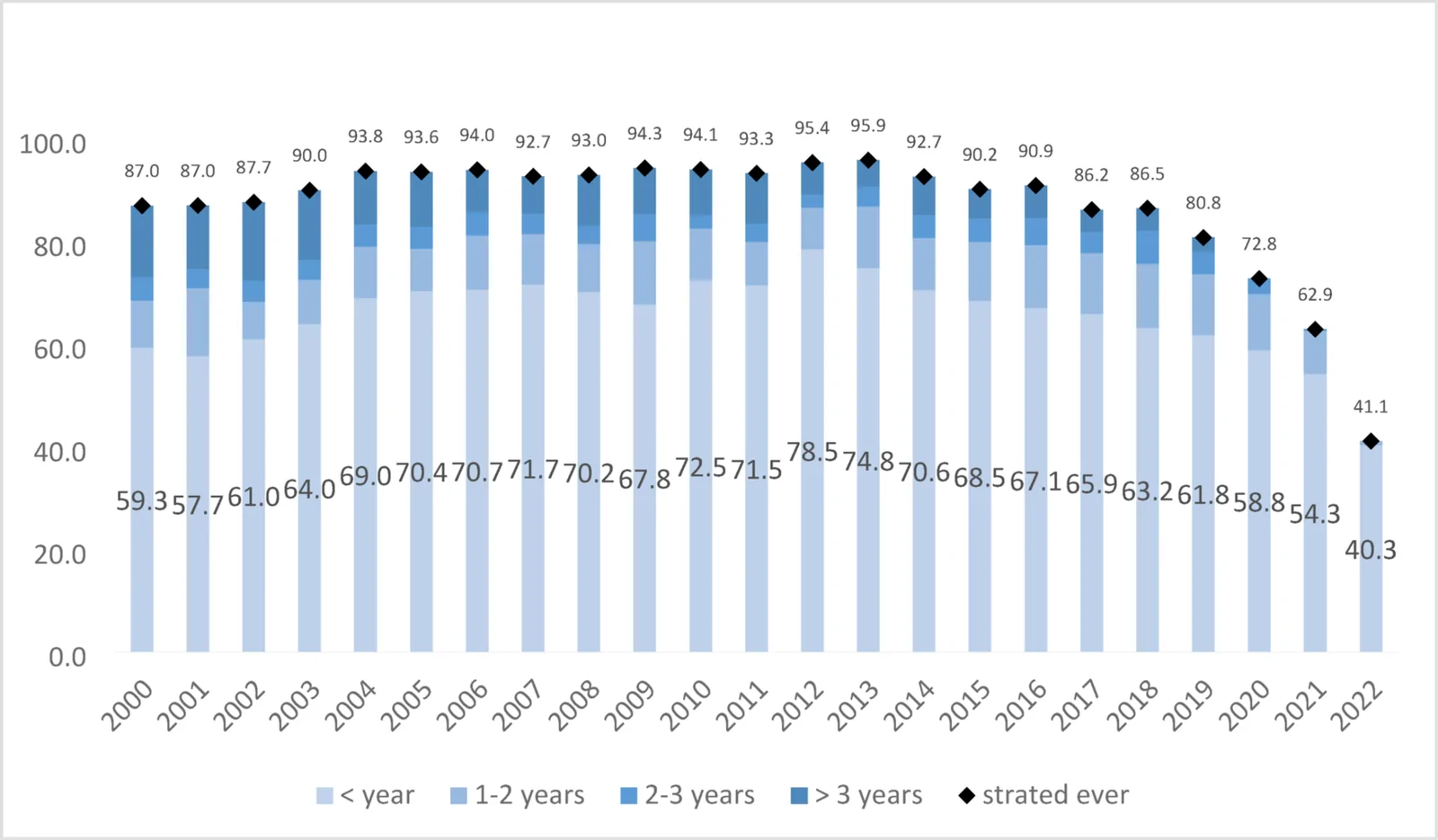
Starting Residency at Any Stage Among All Medical License Holders (Excluding Academic Reserve Program Graduates and New Immigrants), by Year of License Issuance

The number of newly licensed physicians increased significantly over the study period—from 369 in 2000 to 1,622 in 2022. This increase is largely due to higher graduation rates from Israeli medical schools and returnees from foreign institutions. Despite the growth in licensed physicians, no consistent trend was observed in the time elapsed between licensure and the start of residency. While a national database tracking the total number of residency slots does not currently exist, the stability of the residency entry rate through 2019—despite the significant growth in licensed physicians—suggests that total slot availability alone may not fully account for the barriers to entry observed during the study period (Fig. 1).

### Duration until residency start

**Fig. 2:**
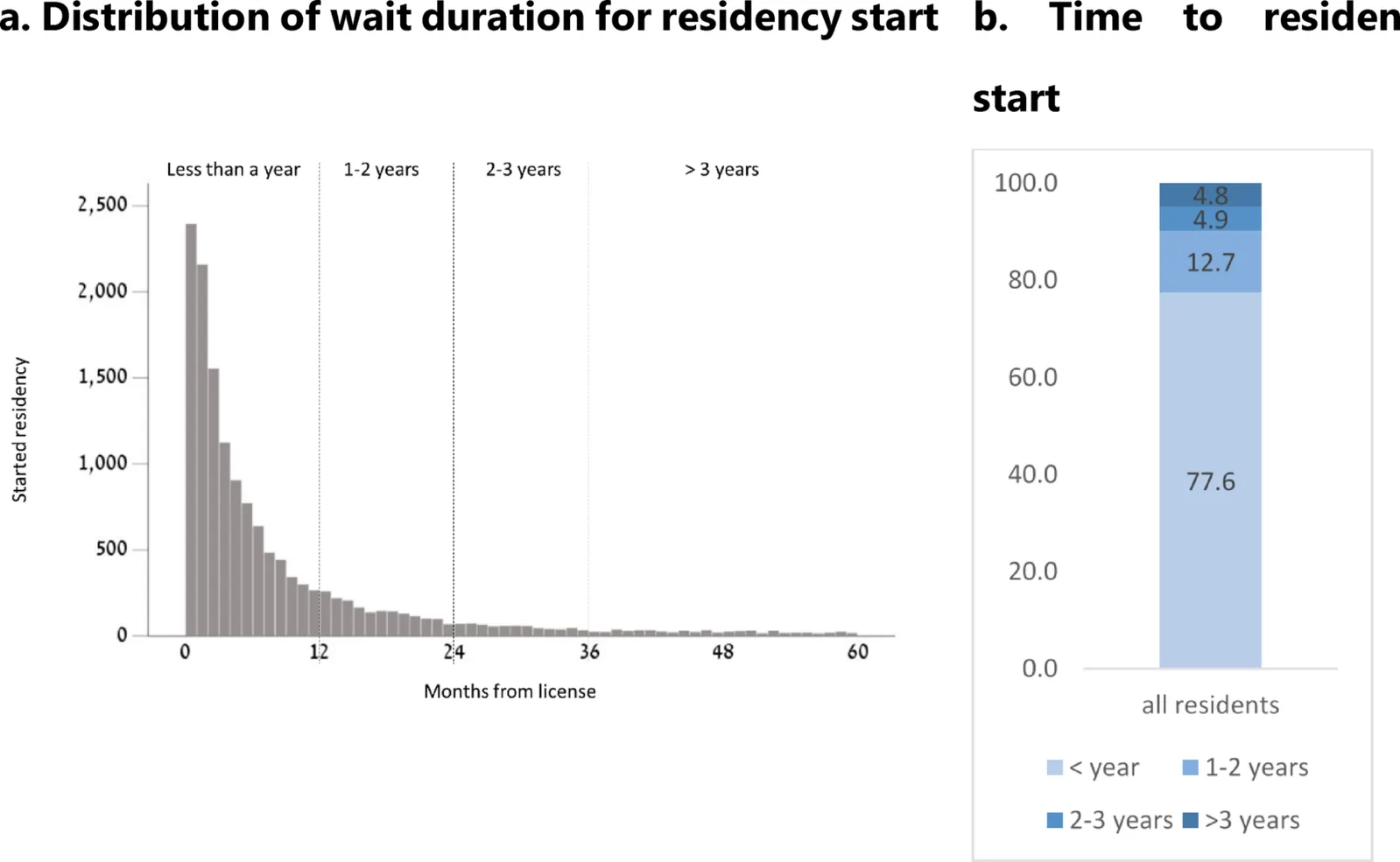
Duration of wait for residency start among residents who began residency within five years of license receipt, 2000–2020

Figure 2 described the time elapsed from license receipt to residency start for all physicians who began residency within five years of receiving their license. Most residents (77.6%) began residency within 12 months of license receipt—the majority within a few months. Only 4.8% of residents began residency after more than three years. The analysis revealed that as years pass after license receipt, the chance of finding a residency position decreases.

### Barriers in the residency admission process

From the interviewees’ statements, it emerges that the residency admission process is not clear to residents. There appears to be no standardized protocol that obligates departments training residents—each hospital and each department manage the process as they see fit. The unique characteristics of medical residency alongside the opaque and inconsistent admission process may cause delays and barriers in the residency admission process, harming both candidates and the health system.

1. **Ambiguity in Department Admission Procedures and Variation in Admission Criteria**

In all hospitals mentioned in the interviews, department heads manage the admission process for residency candidates according to their departments’ needs and residents’ areas of interest, each according to their own independent professional judgment. For example, there are managers who emphasize deep familiarity of candidates with professional knowledge in their field, and others accept candidates based on their desire to be partners in conducting clinical research.

One of the recurring complaints of residency candidates and residents in the interviews was the lack of transparency in the criteria required for residency admission: “There were 15 candidates for this position and no one knew what the criteria were… like, you feel like you don’t have some organized structure that lets you understand why you’re accepted, why you’re not accepted” (Interviewee No. 19, waiting for residency, graduate of domestic studies).

Some department heads decide whether to accept a residency candidate alone after speaking with them, others convene a departmental committee including specialists and residents that discusses the candidate’s suitability based on criteria important to the department head, and there are departments where the department head instituted filling out a sociometric questionnaire by residents in their department about residency candidates.

There are differences between hospitals in the level of involvement of hospital managers and deputy CEOs responsible for human resources in the resident recruitment process. Some senior managers allow department heads to make independent decisions and testify about themselves as merely a ‘rubber stamp’. Others review data on each of the candidates proposed by department heads and sometimes disqualify candidates based on institutional considerations.

A senior manager at a large hospital described the lack of transparency existing in the process—at his institution and in hospitals generally: “The entire resident recruitment issue is a very unregulated matter, lacking rules, lacking explicit guidelines, and there’s really no order to it, incidentally for better and often for worse too” (Interviewee No. 2, hospital deputy CEO).

2. **Order of Position Availability and Their Publication**

Israel’s health system does not have a central system for managing the residency array. Each hospital has its own separate system, including hospitals operating under joint management such as health maintenance organization hospitals. Therefore, the Ministry of Health does not know what the national resident roster is at a given point in time in each field and what stage they are at during residency. Thus, the health system and residents do not have an information source that centralizes the forecast of position availability. Currently, there is no formal mechanism for data sharing between the Scientific Council of the Israel Medical Association and the Ministry of Health. This lack of integration is a significant barrier, as it prevents the Ministry from accessing real-time data on the national residency roster and training vacancies, which is essential for effective workforce planning.

Department heads interviewed in this study testified that they open residency positions according to the expected workload in their department in the coming years, after consultation with hospital managers on the matter. The date when residency positions open is not fixed and usually depends on the completion date of one of the residents’ residency in the department or the opening of a hospital position, so there is no uniformity in position availability and therefore the resident must specifically inquire about the timing of position availability in each department. In the current Israeli system, residency slots are primarily allocated to departments based on clinical workload and staffing needs rather than being determined by independent review boards based on training capacity. This decentralized approach, in which hospital-level and professional stakeholders play a central role, often lacks a broader strategic national planning perspective.

Some candidates described that sometimes the opaque and frustrating process led them to psychological distress and a negative search experience, as they do not know when they should submit applications and therefore may miss an opportunity to specialize in a desired department: “People in this process, because of this ambiguity, get lost both psychologically and professionally… and I don’t get any answer about what’s wrong with me. I just get that I’m not suitable and that’s it. A decree of fate” (Interviewee No. 19, waiting for residency).

3. **The Significance of the Internship Year and Its Impact on Residency Admission Chances**

The current physician training pathway in Israel allows medical students exposure to a limited number of departments and fields during the internship year, and some fields are not defined as mandatory areas. Interviewees emphasized that the internship year may play an important role in shaping future residency trajectories. Interviewees reported that decisions made during this year - especially elective choices - can significantly affect the ability to secure a residency afterward.

Candidates who know which profession they want to specialize in try to reach appropriate departments during the internship, through direct internship^2^ or by choosing “electives” in departments where they will eventually specialize. However, there are quite a few cases where the residency candidate stays as an “elective student” in a department where they think they want to specialize in their future path, but eventually, they do not find their place there after receiving the license. This could be due to several reasons: either due to the department head’s unwillingness to accept them as a resident, or because a residency position does not become available in this department close to receiving their license. In some cases, the residency candidates themselves conclude that this is not the appropriate department for them. In each of these cases, residency applicants may face difficulties in securing a position in a department other than the one where they trained during their internship.

Residency candidates who are not sure which field they want to specialize in may miss a window of opportunities. There are quite a few medical students who, at the internship stage, are not at all sure about the profession or department in which they want to specialize. Graduates who felt they were delayed until residency start claimed that this uncertainty was a significant reason for their delay, and that after reaching a decision about the field they are interested in, the elective period was already behind them, and they had to find alternative ways to present themselves to department heads, usually as external shift workers or in observations.

Negative internship experiences were reported by some participants as contributing to delays. A resident testified that she experienced the internship year as an unpleasant year, and this experience caused her to wait over a year between license receipt and residency start: “I personally came out very, very angry after the internship and had a bad year… The attitude of residents, senior residents, senior physicians toward interns, the management’s perception of interns, everything was very dismissive and unpleasant” (Interviewee No. 14, resident).

### Personal preferences of residency candidates

1. **The Relationship Between Specialty Field and Duration of Wait for Its Start**

**Fig. 3:**
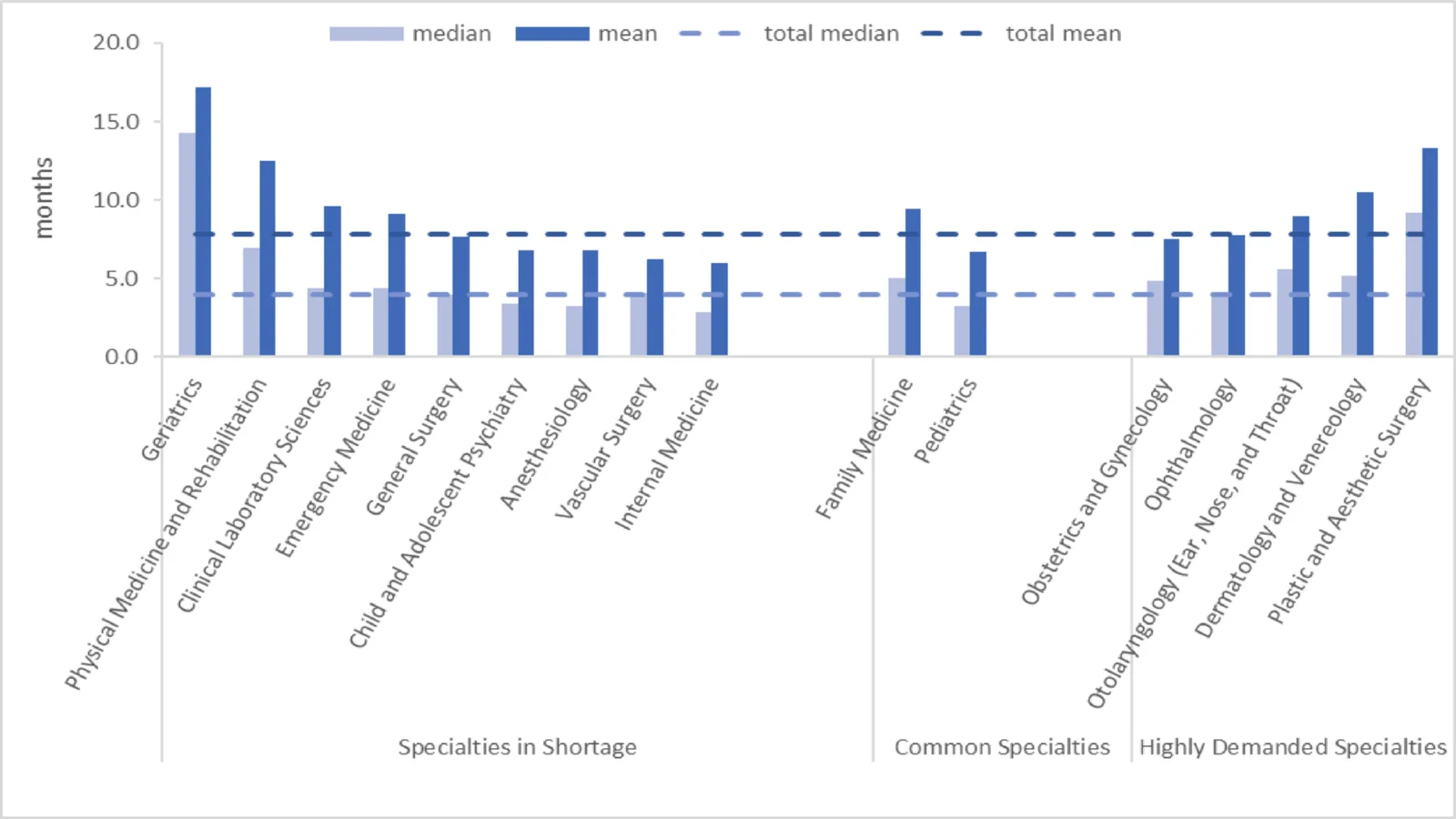
4 Average and median waiting time for residency start, by specialty field, 2010–2022 (in months)

Delays between license receipt and residency start may also stem from personal preferences of residency candidates. One of the factors subject to the residency candidate’s discretion is choosing the specialty field. Figure 3 presents the distribution of waiting time until residency starts by specialty field for several selected fields. The median waiting time is 3.6 months, and the average waiting time is 7 months. The median waiting time until starting residency in internal medicine, the most common residency field, is 3 months, the lowest among all residencies. The average wait for it is 7 months. This likely reflects its popularity, although internal medicine remains a specialty in shortage due to ongoing high demand. The only residencies with slightly shorter waiting times than internal medicine are oncology and neurology^3^ .

Many interviewees estimated that waiting times for residency start are affected by the level of demand for the specialty field. Numerical analysis showed that the relationship between the level of demand for the specialty field and the waiting time for it is not unambiguous. While waiting times until starting residencies in dermatology and plastic and aesthetic surgery, considered very sought-after, are relatively long compared to the overall median and average, long waiting times are also noticeable for several professions defined as suffering from personnel shortages such as geriatrics and rehabilitation. One possible explanation suggested in interviews is that after several months or years of waiting for other residencies, candidates compromise and turn to geriatrics residency, which is easier to get accepted to: “[Interviewer: ] And today are you willing to specialize in anything? [Interviewee: ] Yes. [Interviewer: ] Yes? Even if they tell you geriatrics? [Interviewee: ] Yes. I don’t care, in the end I want a position, I want to be a specialist physician” (Interviewee No. 23, waiting for residency).

Simultaneously, in interviews it was often claimed that many residency candidates are interested in specializing in obstetrics and gynecology, ophthalmology, and ENT, and even delay over a year after license receipt waiting for residency positions to open in these fields. However, from data analysis it appears that waiting times for these fields are very similar to the national average, and that half of those waiting for these fields begin residency within about four months of receiving their license.

2. **The Relationship between Department Location and Resident Placement Dynamics and Quality**

One of the central areas affecting physicians when choosing residency is the size and location of the hospital. A large tertiary hospital in the center of the country is not comparable to a small peripheral hospital in the south or north. Generally, it can be said that hospitals and departments located in the central area, especially in Tel Aviv, enjoy particularly high demand from candidates who want to specialize in them, while peripheral hospitals have noticeable difficulty filling all residency positions in their institutions, especially with residents whose professional level is adequate.

As senior physicians testified: “There are many people who won’t come because we’re periphery… especially in the north, it’s so hard for them to get good residents, it’s very, very difficult” (Interviewee No. 5, department head). “What happens today is that they (in the periphery) don’t even get the less good ones from the center, they get all those who really have no ability to integrate” (Interviewee No. 4, hospital deputy CEO).

Even medical faculties located in the periphery do not provide a solution to the needs of hospitals in the area, and many students return or move to live in the center after studies or after completing the internship, both due to technical difficulties and the need for family assistance in raising children and due to perceptions regarding differences in training level between hospitals: “It was funny, because from my group, I and two other residents remained. And this was considered a significant achievement they talked about, how could it be that from a class of 64 people, having three remain in the north was considered a very big achievement for them… you understand the escape back. Not many stay. One, because again, it’s harder, most people are at ages to have children or small children, need the help. Two, there’s this stigma, and it’s somewhat true, that medicine here is much less—the training here is much less good…” (Interviewee No. 10, resident).

From the interviews, it emerges that managers of central hospitals enjoy a large candidate pool and choose skilled residents, while peripheral managers have been pointing out for years the distress in finding residents and trying to solve it in various ways.

A manager from the periphery suggested that the standard residency track includes exposure to work in departments of more than one medical institution, so that peripheral hospitals would receive the same physicians who specialize in central hospitals:

“… say you do three months in four other hospitals… in our small country, the patients received and the diseases they have are very, very, very different from one medical center to another, so it’s also good for residents to move around in different departments and be exposed, and it’s excellent for hospitals. And then [peripheral hospital] doesn’t need to fight for good residents…” (Interviewee No. 5, department head).

The Ministry of Health incentivizes residents to turn to peripheral hospitals through various programs. This is in the hope that residents will stay in the hospital area where they specialized even after completing residency and starting their work as senior physicians: “Once you leave someone in the residency years and they really settle here and build a family here, it will be much harder for them to return” (Interviewee No. 10, resident).

Another idea that came up in interviews was to try to intervene in the process during medical school admission. One of the senior physicians suggested that peripheral candidates who apply to medical schools in Israel should be prioritized for admission to medical schools in their area of residence, even if their data are slightly less good, looking at medical schools also as a regional resource and not just national. “Those from the south, who have potential, so maybe he has 5 more points on the psychometric, but it’s really meaningless, it’s meaningless, but because of that he wasn’t accepted here to Ben Gurion [a medical school located in the southern region] and then he’ll go to Romania, won’t study properly, will come back here and no one will employ him, so the south fills up with people who studied studies that aren’t worth anything” (Interviewee No. 1, senior manager).

3. **Personal Reasons for Delays in Residency Start**

Internship graduates are usually young people in their late twenties or early thirties, after six years of study (usually) and an intensive internship period. It can be assumed that some internship graduates will choose to take a voluntary break before starting residency, either for rest and vacation or for starting a family. Despite this initial assumption, both the interviews and the quantitative data analysis suggest that there are almost no residency candidates who postpone residency for personal reasons.

## Discussion

This study examined the barriers that delay the start of medical residency in Israel, using both administrative data and stakeholder interviews. While Israel has increased the number of newly licensed physicians in recent years, our findings suggest that systemic inefficiencies and lack of national coordination in residency placement contribute to underutilization of available medical personnel. These delays have real implications: Delays in entering residency have implications for both individual career progression and overall workforce planning.

To quantify the impact of these delays, we consider the career-span of a physician, typically estimated at 35 years or more (420 months). Given the average delay of 8.2 months identified in this study, the system effectively loses approximately 1.95% of a physician’s total productive career due to uncoordinated search processes. While this percentage may seem modest at the individual level, the aggregate impact is substantial: for our study population of 18,480 licensed physicians, the average 8.2-month delay translates into a cumulative loss of 12,628 years of specialist labor. Shortening this interval offers an immediate mechanism to increase workforce capacity by nearly 2% without the long lead times required for expanding medical school enrollment.

Beginning in September 2025, the internship period (stage) in Israel was shortened from twelve months to nine months. In addition, modifications were made to the structure and duration of the various clinical rotations in order to enhance the quality of training. A further reduction is expected in 2027, which in certain cases will allow the internship to be shortened to as little as six months. The reform was driven by the need to streamline the internship period, thereby expediting the professional maturation of medical graduates and facilitating their earlier transition into residency and, subsequently, into specialist roles within Israel’s healthcare system (Ministry of Health, [6]).

This study identified several major barriers to the optimal utilization of the existing potential of candidates to residencies and to its efficient allocation across professions and regions.

### Delays are not due to personal choice

Contrary to common assumptions, most graduates do not intentionally delay their entry into residency. The quantitative data show that only a small proportion postpone residency beyond one year, and qualitative interviews revealed that when such delays occur, they are typically due to systemic obstacles, not personal decisions. This challenges the idea that post-internship “gaps” are a lifestyle choice and highlights the need for structural reform.

### Opaque and fragmented residency admission process

The most prominent finding is the lack of a transparent and standardized admission process. Residency candidates in Israel must individually contact departments, often without knowing whether a position is available, what selection criteria are applied, or who makes the final decision. This creates unnecessary psychological distress, wasted effort, and inconsistent outcomes. The situation is especially problematic for international graduates, candidates without strong local networks, and those applying to highly sought-after specialties.

### Lack of national data transparency

In addition to the lack of transparency for the candidates, there is a lack of transparency within the healthcare system.

The findings of this study reveal a profound lack of coordination among key stakeholders. Currently, the transition from licensure to residency is managed by multiple players—the Ministry of Health, the Ministry of Finance, the Scientific Council, and individual department heads—each operating within separate silos with misaligned incentives. This fragmented structure is not merely a background condition but the root cause of the observed inefficiencies. Without a centralized framework to synchronize these actors, any attempts to reform will remain fragmented. Our proposed solution is to better integrate these disparate elements into a single, transparent marketplace.

### Lack of national planning

One of the most significant structural gaps is the absence of a national residency matching system. Israel relies on an entirely decentralized and informal process, in stark contrast to countries that have adopted coordinated national mechanisms. In the United States, the National Resident Matching Program (NRMP) uses an algorithmic matching system to assign candidates to programs based on mutual ranking preferences. All results are published on the same date, reducing anxiety and speculation. In Canada, the Canadian Resident Matching Service (CaRMS) [1] serves a similar function, promoting fairness, transparency, and access to detailed statistics on match rates and program demand. In Spain, the Médico Interno Residente (MIR) exam ranks all residency candidates nationwide. Placement is based on exam score and declared preferences, ensuring clarity about admission likelihood and availability.

These systems not only enhance fairness and transparency, but also support national workforce planning, allowing governments to adjust specialty quotas, track vacancies, and identify underserved areas.

### Internship year: implications of limited clinical exposure

Internship experiences, especially elective rotations, play a key role in shaping future residency outcomes. Candidates who are unsure of their specialty or who have negative internship experiences could have benefited from a valuable opportunity establishing connections or demonstrating commitment. While the internship year does not necessarily determine the ultimate likelihood of entering residency, it significantly influences the timing of commencement. A high-quality internship, particularly through strategic elective choices, facilitates earlier career clarity and professional networking, thereby accelerating the transition into residency.

Both the internship format that was in place until 2026 and the revised model, which includes rotations in four core disciplines and two months of elective training, do not provide exposure to the full spectrum of relevant specialties. Consequently, they offer only limited support for trainees who remain undecided regarding their choice of residency.

More fundamentally, this limits the total number of positions to which candidates will apply - they apply where they completed their rotations and neither see nor become familiar with all other available sites, thereby restricting market thickness - that is, the number of effective interactions between candidates and available positions - and perpetuating information asymmetries between candidates and departments.

However, the decision not to expand elective rotations reflects the need to keep the internship at a manageable length and enable timely entry of new physicians into the system. Given the importance of electives, their number and duration were maintained in the 2026 reform, reflecting an attempt to balance informed specialty choice with system-wide planning needs.

### Geographic disparities threaten workforce equity

Hospitals in the geographic periphery face ongoing challenges in recruiting qualified residents. While residency programs in central hospitals enjoy high demand and applicant selectivity, peripheral institutions often struggle to fill all available positions. This maldistribution perpetuates inequalities in health workforce availability and potentially in quality of care across regions. Enhancing the attractiveness and training quality of peripheral residency programs is a critical lever for reducing overall national delays. By redistributing demand more evenly across the country, the healthcare system can mitigate the ‘bottleneck’ in high-demand central hospitals and shorten the wait times for candidates who otherwise remain in prolonged search processes.

In recent years, several significant initiatives have been advanced to attract and retain high-quality personnel within the healthcare system. These include a substantial increase in the number of medical students (the *“Ilanot”* program; Ministry of Health) and, to a lesser extent, an expansion in the number of medical residents (the *“Kochavim”* program Ministry of Health) [5, 6]. Another initiative currently being advanced is “Ofakim” program which offers generous scholarships to outstanding students who have studied abroad, in exchange for a commitment to return and complete their residency training in hospitals located in the periphery [7]. However, the number of residents remains relatively limited in comparison to the existing gap, and in the case of medical students, it will take several more years before these efforts are reflected in a substantial rise in the potential pool of residents.

### Policy recommendations

Based on the findings of this study, several actionable steps can be taken to reduce delays in residency placement and improve the efficiency, equity, and transparency of the Israeli residency system. The set of recommendations presented below is intended to address structural barriers in the transition from licensure to residency. Taken together, these measures aim to facilitate a more transparent, structured, and accessible process, enabling candidates to identify and secure residency positions more efficiently. By reducing uncertainty and the burden placed on individual candidates in navigating the system, these changes may shorten the time from licensure to residency entry. At the system level, this is expected to support a more timely and efficient integration of physicians into residency training, thereby improving the effective utilization of the existing workforce.

Implementation of reform in this area is inherently complex, as it requires coordination among multiple stakeholders whose institutional roles, incentives, and priorities do not always align. While this article includes policy recommendations, its primary purpose is analytical: to identify structural barriers and outline evidence-based directions for reform, rather than to present a formal governmental implementation plan.

### Increase transparency of admission criteria and dates

Departments and hospitals should be required to publish basic eligibility requirements, preferred candidate attributes (e.g., prior experience, research interest), contact points for applications, and dates of anticipated availability. Standardizing appointment decisions even if actual start dates vary would prevent unraveling, increase transparency, and enable candidates to make informed decisions at a defined point rather than navigating continuous interviews. This would help candidates reduce confusion and improve trust in the process.

### Publicize real-time availability of residency positions

A national dashboard should display open residency positions by specialty and hospital, estimated start dates, and length and structure of each program. Such visibility would significantly reduce wasted effort and waiting times, and better align candidate interest with institutional needs.

### Establish a centralized national data system

The foremost priority for the Israeli healthcare system must be the assembly and publication of accurate, annual data on the physician workforce. The current detachment between the Ministry of Health and the Scientific Council must be bridged by a better coordinated healthcare system. This coordination will create a data system which will resolve the systemic ‘congestion’ and low ‘market thickness’ [15] issues currently plaguing the transition from licensure to residency.

### Establish a centralized national residency matching system

Israel should consider developing a national residency allocation system, modeled after successful frameworks such as the NRMP (USA), CaRMS (Canada), or MIR (Spain). Such a system would provide a centralized database of open residency positions, allow for simultaneous and structured applications, use objective matching criteria to reduce bias and uncertainty, might improve equity for applicants without personal connections, prevent unraveling and improve market stability and the matching process [15]. Centralized residency matching systems may contribute to national medical workforce planning by generating standardized, comprehensive data on applicant flows, specialty preferences, and training capacity [10]. Thus, it may also enable better workforce planning and forecasting at the national level. While full implementation may take time, phased pilots in selected specialties or regions could be an effective starting point.

### Enhance clinical exposure and internship structure to support informed career decisions

Medical students should receive expanded exposure to diverse specialties during their clinical training years through structured rotations and elective opportunities, enabling them to explore various fields before committing to a specialty choice. In light of this, even in the new and shortened internship format, the two-month elective period has not been reduced. Internship rotations should be restructured to ensure broad exposure to major specialties before elective choices, provide structured career counseling and mentorship, and clarify whether “direct internships” (those counting toward future residency) exist and how to access them. This would help students make more informed specialty and site selections and reduce regret-driven reapplications. It is important to note that the very introduction of transparency and the ability to make informed decisions are expected to improve the process and shorten waiting times — even without centralized matching system, as is commonly practiced, for example, in the United States.

### Strengthening residency programs in underserved settings and specialties

To address workforce shortages in peripheral hospitals and medical specialties in need, the government should incentivize two strategic approaches: (1) establishing dual-center residency programs that join peripheral medical centers with more centralized institutions, and (2) developing combined residency programs that integrate a specialty in need with a more competitive field. Additional measures can be prioritizing admission of local candidates into regional medical schools, and considering return-of-service agreements for scholarship recipients. While some collaborative arrangements between peripheral and central hospital departments already exist, these initiatives remain limited in scope; it is essential to sustain existing partnerships while significantly expanding their scale and reach to improve training quality, enhance recruitment, and promote more equitable healthcare workforce distribution. In the long term, a voluntary “residency map” outlining the preferred allocation of residents across specialties and regions should be considered.

To summarize the recommendation section, while these measures alone are not expected to fully address physician shortages, they may help improve the efficiency of workforce allocation and the effective utilization of available physicians.

In general, measures aimed at improving transparency, information sharing, and publication of admission criteria may be more immediately actionable, whereas the development of a centralized matching mechanism would likely require longer-term institutional coordination and phased implementation.

### Study limitations

Several limitations should be acknowledged. Approximately 18% of licensed physicians did not enter residency during the study period. While some may be practicing as general practitioners or have pursued residency abroad, available administrative data do not allow for a clear characterization of this group. Characterizing this potential workforce remains a priority for future survey-based research and would be important also for strengthening physician workforce planning. The actual residency start rate may be higher than documented—there are license recipients who work in fields that do not require residency, choose residency abroad, or choose voluntarily to leave the profession. Since there is no information about these physici ans, they are defined as those who did not start residency and artificially increase the rate of non-starters.

This study did not track long-term attrition or specialty switching. Future research should investigate these patterns to assess their impact on system efficiency.An important component in the residency selection process is the hospital where the residency will take place, influenced by various considerations such as prestige and proximity to place of residence. This study did not examine the relationship between residency institutions and waiting time for residency because the rate of missing information in the data file is high and does not allow comparison between institutions.

Information about license recipients’ residential addresses was not used because this information was collected close to the file creation date. It’s possible that the recorded address is a result of moving to a residence close to the residency location and not the cause for choosing the residency initially, or perhaps this residential address became current only many years after residency completion.

Because the qualitative sample was recruited through snowball sampling, it may be subject to selection bias, as participants are more likely to refer colleagues with similar experiences or views, limiting the diversity of perspectives represented in the interview data. Nevertheless, the sample included interviewees from diverse backgrounds, institutions, and stages of training, which helps mitigate - but does not eliminate - this limitation.

## Conclusions

The transition from medical licensure to the start of residency training in Israel is often experienced as uncertain, inefficient, and systemic opacity. Although most newly licensed physicians do eventually begin residency, the path is often delayed, mainly for several residency fields, particularly for those who lack strong departmental connections or who apply to competitive specialties and central hospitals. These delays appear to diminish the effectiveness of the health workforce and impose psychological and logistical burdens on early-career physicians.

Our findings indicate that these delays are primarily structural, not personal, and can be mitigated through thoughtful reform. Drawing on lessons from established residency matching systems abroad, Israel has the opportunity to develop a more equitable, transparent, and data-driven residency placement process. A structured, nationally coordinated allocation mechanism would address the practical inefficiencies documented here while aligning with Roth’s framework for effective market design. Doing so will not only benefit future physicians but also strengthen the national health system in its ability to respond to growing demographic and geographic needs.

The shortage of medical personnel could be reduced if the duration between receiving an Israeli medical license and the actual start of residency training were shortened. Reducing this time would probably decrease the shortage of medical personnel because it would extend the time physicians are within the system. Reducing unnecessary delays may improve retention into specialty training and strengthen long-term workforce stability. Moreover, reform must address both the optimization of resident potential and the efficient allocation of physicians across specialties and geographic regions, ensuring that workforce distribution aligns with system-wide needs.

Above all, the findings underscore the need for a robust, transparent, and regularly updated national database on the physician workforce, developed jointly by the Ministry of Health and the Scientific Council. Such a database is a prerequisite for evidence-informed workforce planning, more efficient allocation of residents across specialties and regions, and a more coordinated transition from licensure to residency.

## Data Availability

The dataset used and analyzed during the current study is available from the corresponding author upon reasonable request.

## Footnotes

1 This article does not address the accreditation of foreign medical schools. This matter will be addressed in depth in another article.

2 A long internship period in a specific department considered part of the residency period.

3 data not presented, see full report at https://brookdale.jdc.org.il/publication/delays-in-the-start-of-residency-for-physicians-after-receipt-of-an-israeli-medical-license/.

4 The difference between the mean and the median reflects a right-skewed distribution. From a health policy perspective, the median offers a more accurate representation of the situation.
